# New Solid Solution and Phase Equilibria in the Subsolidus Area of the Three-Component CuO–V_2_O_5_–Ta_2_O_5_ Oxide System

**DOI:** 10.3390/ma15010232

**Published:** 2021-12-29

**Authors:** Grażyna Dąbrowska, Elżbieta Filipek, Piotr Tabero

**Affiliations:** Department of Inorganic and Analytical Chemistry, Faculty of Chemical Technology and Engineering, West Pomeranian University of Technology in Szczecin, Piastow Ave. 42, 71-065 Szczecin, Poland; elafil@zut.edu.pl (E.F.); ptab@zut.edu.pl (P.T.)

**Keywords:** CuTa_2−x_V_x_O_6_ solid solution, phase equilibria, DTA, XRD, semiconductor

## Abstract

The results of the study of the three-component system of CuO–V_2_O_5_–Ta_2_O_5_ oxides showed, inter alia, that in the air atmosphere in one of its cross-sections, i.e., in the CuV_2_O_6_–CuTa_2_O_6_ system, a new substitutional solid solution with the general formula CuTa_2−x_V_x_O_6_ and homogeneity range for x > 0.0 and x ≤ 0.3 is formed. The influence of the degree of incorporation of V^5+^ ions into the CuTa_2_O_6_ crystal lattice in place of Ta^5+^ ions on the unit cell volume, thermal stability and IR spectra of the obtained solid solution was determined. Moreover, the value of the band gap energy of the CuTa_2−x_V_x_O_6_ solid solution was estimated in the range of 0.0 < x ≤ 0.3, and on this basis, the new solid solution was classified as a semiconductor. On the basis of the research results, the studied system of CuO–V_2_O_5_–Ta_2_O_5_ oxides was also divided into 12 subsidiary subsystems.

## 1. Introduction

The three-component system of metal oxides CuO–V_2_O_5_–Ta_2_O_5_ has not been studied so far in terms of the phases formed in it, and also in order to determine the phase equilibria established in this system over the entire range of concentrations of components in the air atmosphere in the solid state. In turn, the CuO, V_2_O_5_ and Ta_2_O_5_ oxides, which are the components of the system selected for the study, belong to an interesting class of compounds, both in terms of their crystal structure and physicochemical properties. These oxides, due to their electrical and optical properties, are commercially important materials that are used in optoelectronic devices, energy conversion and as catalysts or photocatalysts [1,2,3,4,5,6,7,8,9,10,11,12]. Copper (II) oxide is used to obtain optical switches, field emission devices, lithium-ion electrode materials, gas sensors, biosensors and magnetic data carriers [1,2,3,4]. The applications of vanadium (V) oxide include catalytic processes and the production of field-effect transistors, gas sensors, infrared detectors, glass dyes [5,6,7,8,9,10]. Tantalum (V) oxide, due to its high dielectric constant, is used in the production of semiconductors used in DRAM memories, high-frequency CMOS integrated circuits and flash memories [11,12].

The literature data show that the reactivity of oxides in the binary CuO–V_2_O_5_, CuO–Ta_2_O_5_, V_2_O_5_–Ta_2_O_5_ systems constituting a side limitation of the three-component CuO–V_2_O_5_–Ta_2_O_5_ system was the subject of numerous studies. Most of the works concern the CuO–V_2_O_5_ system. They show that in the reactions between CuO and V_2_O_5_ oxides, the following compounds are formed in the air atmosphere: CuV_2_O_6_, Cu_2_V_2_O_7_, Cu_3_V_2_O_8_, Cu_5_V_2_O_10_ and Cu_11_V_6_O_26_ [13,14,15,16,17,18,19,20,21,22,23,24,25,26,27,28]. The basic physicochemical properties of these phases and their thermal stability are known [13,14,15,16,17,18,19,20,21,22,23,24,25,26,27,28]. Copper (II) metavanadate (V) has two polymorphic forms: the α-CuV_2_O_6_ form crystallizing in a triclinic system and the β-CuV_2_O_6_ form crystallizing in a monoclinic system [13,14]. The CuV_2_O_6_ compound melts incongruently at 650 °C with the release of α-Cu_2_V_2_O_7_ [15,16]. The Cu_2_V_2_O_7_ compound crystallizes in three polymorphs: orthorhombic α-Cu_2_V_2_O_7_ [17], monoclinic β-Cu_2_V_2_O_7_ [18] and triclinic γ-Cu_2_V_2_O_7_ [19]. The transition temperature of α→β-Cu_2_V_2_O_7_ is 712 °C [20]. The Cu_2_V_2_O_7_ compound melts congruently at 760 °C [15,21]. Cu_3_V_2_O_8_ has two polymorphs: triclinic α and monoclinic β [22,23] and is stable up to the temperature of 780 °C, where it melts incongruently with the release of Cu_5_V_2_O_10_ [20]. The compounds Cu_5_V_2_O_10_ and Cu_11_V_6_O_26_ do not have polymorphs [24,25,26]. Cu_11_V_6_O_26_ melts incongruently at 795 °C with the release of Cu_5_V_2_O_10_ [15]. Whereas Cu_5_V_2_O_10_ melts incongruently at 820 °C with the release of CuO [26]. Vanadates (V) of copper (II), and especially CuV_2_O_6_, are used as cathode materials in lithium batteries, as catalysts for SO_3_ decomposition during thermochemical reactions of hydrogen production, and as photocatalysts for water decomposition [27,28,29].

Less work has been conducted on the V_2_O_5_–Ta_2_O_5_ system [30,31,32,33,34,35,36,37]. It was found that vanadium (V) oxide reacts with tantalum (V) oxide in the air atmosphere with the formation of TaVO_5_ and Ta_9_VO_25_ compounds [30,31,32,33] and a substitution solid solution with a limited range of homogeneity with the structure of Ta_9_VO_25_, the existence of which was indicated only in work [34]. The TaVO_5_ compound has two polymorphs: orthorhombic and tetragonal [30,31,33]. The temperature of this transformation, depending on the source, is 880 °C [30] or 600 °C [33]. TaVO_5_ decomposes at 940 °C to Ta_9_VO_25_ and V_2_O_5_ [30]. Ta_9_VO_25_ is stable in the air atmosphere at least 1650 °C [31].

It is known from the available literature that the CuTa_2_O_6_ compound is formed in the binary system of CuO-Ta_2_O_5_ oxides [38,39,40,41]. Three crystallographic forms of CuTa_2_O_6_ are known: monoclinic, cubic and tetragonal [38,39,40]. Vincent and his colleagues report that there is also an orthorhombic form of the CuTa_2_O_6_ compound [41]. The temperature of transition of polymorphic monoclinic to tetragonal variety is 227 °C [38]. Due to its properties, the CuTa_2_O_6_ compound improves the piezoelectric properties of ceramics used in the production of transformers, transducers, ultrasonic motors and devices for surface acoustic waves [42,43,44].

The large variety of properties of the oxides that build the CuO–V_2_O_5_–Ta_2_O_5_ ternary system, as well as the compounds formed in its side systems, such as CuTa_2_O_6_ and CuV_2_O_6_, make them interesting for technical applications. Therefore, it seemed justified to study the mutual reactivity of both the oxides and the known compounds in order to find out whether they react with the formation of new phases with potential, interesting application properties.

The presented work also includes the results of research on phase equilibria established in the title three-component system of metal oxides in the entire range of concentrations of the components of this system. The results of these studies allowed us to determine, inter alia, the ranges of concentrations of the components of the tested system and the temperatures in which the identified phases (both known and obtained for the first time in this work) coexist in the solid state.

## 2. Materials and Methods

The following oxides were used for the tests: CuO p.a. (Aldrich, St. Louis, MO, USA), V_2_O_5_ p.a. (POCh, Gliwice, Poland) and Ta_2_O_5_ p.a. (Aldrich, St. Louis, MO, USA). The reactants were weighed in the appropriate amounts, homogenized by trituration, pelleted and heated in an air atmosphere under conditions that allowed the reaction to proceed in the solid phase. The samples were heated in the temperature range of 550–900 °C in several 24-h stages.

After each heating stage, the samples were left in the furnace until they cooled to room temperature and then were ground and tested by XRD to determine their phase composition.

The temperatures of the final stage of heating the samples and the kind of phases in equilibria are given in Table 1 and Table 2.

The powder diffractograms of the tested phases were recorded at room temperature using an Empyrean II diffractometer (CuKα radiation, graphite monochromator, semiconductor PIXcel3D detector, measuring step 0.013°, counting time in the measuring interval 70s, manufacturer PanAnalytical, The Netherlands). The phases were identified using the data from the ICDD 2021 PDF database. The Refinement program (DHN, Poland) was used to indexation the powder diffraction patterns.

DTA-TG studies were performed with the F. Paulik–L. Paulik–L. Erdey derivatograph, MOM Budapest, Hungary. The measurements were made in the air atmosphere, in the temperature range 20–1000 °C, with the galvanometer’s sensitivity of DTA 1/5 and the constant heating rate of 10 °C/min. All tests were performed in quartz crucibles. The mass of the tested samples was always 500 mg. The accuracy of the temperature reading, determined on the basis of iterations, was found to be ±5 °C.

Selected samples were tested by the DTA-TG method using the SDT 2960 from TA Instruments. Measurements were carried out in an air atmosphere, in the temperature range of 20–1500 °C, at a heating rate of 10 °C/min. Measurements were made in corundum crucibles. The mass of the tested samples was ~20 mg.

The IR spectra were recorded with the help of the SPECORD M 80 IR spectrometer, manufactured by Carl Zeiss, Jena, East Germany, using the KBr pellet pressing technique in a mass ratio of 1:300. Measurements were made in the range of wavenumber 1200–200 cm^−1^.

A UV-Vis-V-670 spectrophotometer (JASCO, Tokyo, Japan) with an integrating sphere PIV-756/PIN-757 was used for the UV-Vis spectroscopy tests. The optical absorption was measured in the 200–1000 nm range at room temperature.

As part of the presented work, tests of phase equilibria in the CuO–V_2_O_5_–Ta_2_O_5_ system were additionally carried out in the air atmosphere in a solid state. In this stage of the study, 20 samples made of oxides were synthesized in conditions ensuring achievement of the equilibrium state, and the achievement of such a state was found when the results of the samples, carried out with the XRD and DTA methods after two successive heating stages, were identical, and the number of phases was consistent with the extended Gibbs phase rule.

## 3. Results and Discussion

### 3.1. Reactivity of CuO and V_2_O_5_ with Ta_2_O_5_ in the Air

The study of the three-component system of CuO–Ta_2_O_5_–V_2_O_5_ oxides began with the examination of one of the hypothetical cross-sections of this system, i.e., CuV_2_O_6_–CuTa_2_O_6_. For this purpose, ten samples were prepared from the separately obtained compounds, i.e., CuTa_2_O_6_ and CuV_2_O_6_, with compositions selected to represent the entire range of concentrations of the components of the tested system (Table 1). The location of weighed samples′ compositions is marked in the area of the triangle of concentrations of the components of the CuO–Ta_2_O_5_–V_2_O_5_ system.

The synthesis of the compound CuTa_2_O_6_ was carried out in an air atmosphere according to the reaction equation below:

Ta_2_O_5(s)_ + CuO_(s)_ = CuTa_2_O_6(s)_ (1)

Under the following conditions: 900 °C (24 h) → 1000 °C (24 h) → 1050 °C (24 h) → 1200 °C (24 h). The obtained compound showed a green color, and the position of the lines in the diffraction pattern was consistent with the data given in the PDF card 00-032-0349, which proved that the tetragonal form of the CuTa_2_O_6_ compound was obtained.

In order to obtain copper (II) metavanadate (V), the sample consisting of oxides with the composition: 50 mol% CuO and 50 mol% V_2_O_5_ was weighed and subjected to two-stage 24-h heating at the following temperatures: 550 °C and 600 °C. A plum gray compound was obtained. X-ray phase analysis of the sample after the last heating step confirmed that the diffraction lines characterizing this compound are consistent with the data contained in the PDF card of the triclinic modification polymorph of the CuV_2_O_6_ compound with the number 00-045-1054, i.e., α-CuV_2_O_6_.

The results of the XRD analysis of the tested samples after the last heating stage and the temperature of the last heating stage are presented in Table 1.

The phase compositions of samples No. 1 and No. 2 in the equilibrium state presented in Table 1 and contained in the initial mixtures were 90.00 mol% CuV_2_O_6_ and 10.00 mol% CuTa_2_O_6_, and 75.00 mol% CuTa_2_O_6_ and 25.00 mol% CuV_2_O_6_, respectively, indicating that the samples reacted as:

3 CuV_2_O_6(s)_ + CuTa_2_O_6(s)_ = 2 Cu_2_V_2_O_7(s)_ + 2 TaVO_5(s)_ (2)

In sample 1, the components CuV_2_O_6_ and CuTa_2_O_6_ in the reaction mixture remained in a molar ratio of 9:1; therefore, with respect to the stoichiometry of the reaction taking place in the reaction mixture, the CuV_2_O_6_ substrate is in excess and therefore, this compound is ultimately in equilibrium with the reaction products (2).

The phase composition of samples 3 and 4 at equilibrium shows that the CuV_2_O_6_ present in the samples reacted with CuTa_2_O_6_ with the formation of three compounds Cu_2_V_2_O_7_, TaVO_5_ and Ta_9_VO_25_. Thus, in sample No. 3, the initial mixture of which contained 66.67 mol% of CuV_2_O_6_ and 33.33 mol% CuTa_2_O_6_, the reaction proceeded according to the following reaction equation:

16 CuV_2_O_6(s)_ + 8 CuTa_2_O_6(s)_ = 12 Cu_2_V_2_O_7(s)_ + 7 TaVO_5(s)_ + Ta_9_VO_25(s)_ (3)

The presented results of the X-ray analysis of samples 5–9 after the last heating stage show that in these samples, the reaction between CuV_2_O_6_ and CuTa_2_O_6_ resulted in the formation of compounds, i.e., Cu_2_V_2_O_7_ and Ta_9_VO_25_. The excess substrate in these reactions is CuTa_2_O_6_. For sample No. 7, which in the initial mixture contained 40.00% mol of CuV_2_O_6_ and 60.00% mol of CuTa_2_O_6_, the reaction proceeded according to the reaction equation:

11 CuV_2_O_6(s)_ + 9 CuTa_2_O_6(s)_ = 10 Cu_2_V_2_O_7(s)_ + 2 Ta_9_VO_25(s)_ (4)

Heating of sample 10 at the temperature of 900 °C resulted in the presence of only lines characterizing the compound CuTa_2_O_6_ in the diffraction pattern. These lines were slightly shifted towards the higher angles 2θ, i.e., they corresponded to smaller values of the d_hkl_ interplanar distances. It was found that the shift of the diffraction lines belonging to the CuTa_2_O_6_ set and the absence of the Cu_2_V_2_O_7_ compound in the sample proves that a substitutional solid solution is formed in the sample, the matrix of which is CuTa_2_O_6_, according to the reaction equation:

(1 − 0.5x)·CuTa_2_O_6(s)_ + 0.5x·CuV_2_O_6(s)_ = CuTa_2−x_V_x_O_6(s.s.)_ (5)

In order to determine the homogeneity range of the formed solid solution, additional samples were prepared from the finished phases, i.e., CuTa_2_O_6_ and CuV_2_O_6_, containing 12.5 mol%, 15.00 mol% and 17.5 mol% CuV_2_O_6_, respectively, in the initial mixtures. X-ray phase analysis showed that only the samples with the initial composition of 12.5 mol% CuV_2_O_6_ and 87.50 mol% CuTa_2_O_6_ and 15.00 mol% CuV_2_O_6_ and 85 mol% CuTa_2_O_6_ were monophasic and contained CuTa_1.75_V_0.25_O_6_ and CuTa_1.70_V_0.30_O_6_ solid solution, respectively.

The chemical formula of the formed solid solution was determined on the basis of the composition of the initial mixture of substrates, taking into account their complete conversion, which was found on the basis of the X-ray phase analysis of the synthesized samples after the last stage of heating them at 900 °C (Table 2).

In the sample whose initial mixtures contained 17.50 mol% CuV_2_O_6_ and 82.50 mol% CuTa_2_O_6_, the phases: CuTa_1.70_V_0.30_O_6_, Cu_2_V_2_O_7_ and Ta_9_VO_25_ were present at equilibrium.

In Table 2, apart from the composition of the initial mixtures of substrates from which single-phase samples containing a solid solution were obtained, the phase composition of the sample after exceeding its homogeneity is given.

At this stage of the research, however, it cannot be ruled out that in the obtained single-phase samples containing the solid solution, no other phases are still present, but if so, they are in amounts not detectable by the XRD method. However, this does not affect the general formula of the obtained new solid solution and the proposed range of its homogeneity.

Based on the research, it was found that the CuTa_2−x_V_x_O_6(s.s.)_ solid solution formed in the three-component CuO–V_2_O_5_–Ta_2_O_5_ oxide system has a homogeneity range of 0 < x ≤ 0.3, which shows that the maximum degree of incorporation of V^5+^ ions in place of ions Ta^5+^ in the CuTa_2_O_6_ crystal lattice is 15 mol%.

As part of the work, samples containing a CuTa_2−x_V_x_O_6_ solid solution, where x = 0.30 in equilibrium with other phases, were annealed at higher temperatures and then frozen (rapidly cooled to room temperature) in order to determine the change in their phase composition. XRD analysis did not show such a change, which means that the solubility range of the synthesized solution does not significantly depend on the temperature.

**Figure 1:**
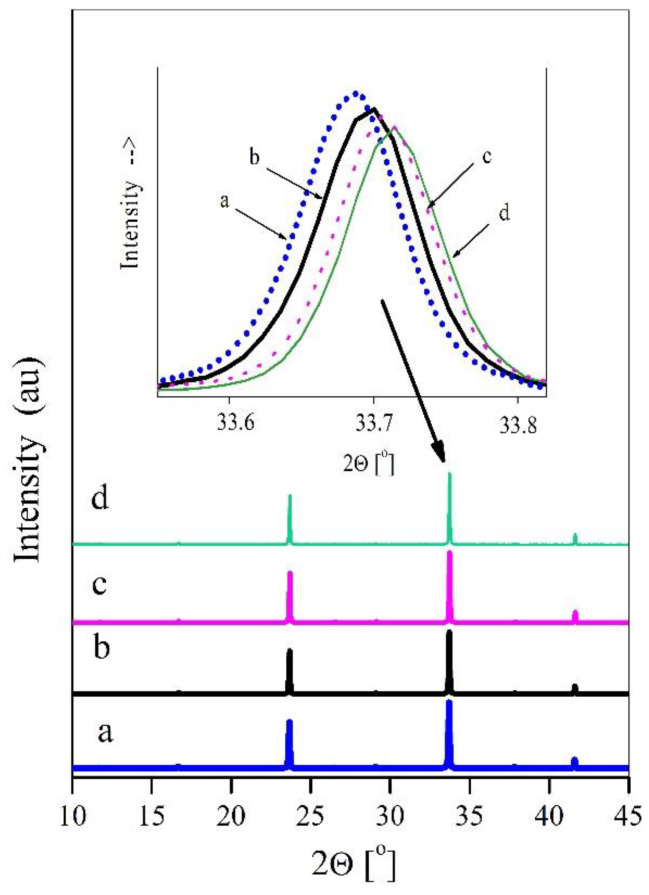
Fragments of X-ray diffraction patterns of: (**a**) CuTa_2_O_6_ (x = 0), (**b**) CuTa_1.80_V_0.20_O_6_ (x = 0.20), (**c**) CuTa_1.75_V_0.25_O_6_ (x = 0.25), (**d**) CuTa_1.70_V_0.30_O_6_ (x = 0.30).

Figure 1 presents fragments of the powder diffraction patterns of the CuTa_2_O_6_ compound (fragment a) and the CuTa_2-x_V_x_O_6_ solid solution for x = 0.20; 0.25 and 0.30 (fragments b–d) showing changes in the angular positions of selected diffraction reflections with an increase in vanadium content in CuTa_2−x_V_x_O_6_ samples. The analysis of the presented diffractograms showed that with the increase of the degree of incorporation of vanadium ions into the crystal lattice of the CuTa_2_O_6_ matrix, the diffraction reflections shifted towards higher 2θ angles up to x = 0.30.

Indexing of the powder diffraction patterns of the CuTa_2−x_V_x_O_6_ solid solution for x = 0.20; 0.25 and 0.30 and the CuTa_2_O_6_ compound were made using the REFINEMENT program. The results of the indexing, presented in Table 3, indicate that with the increase of the degree of incorporation of V^5+^ ions in place of Ta^5+^ ions into the CuTa_2_O_6_ crystal lattice, the a = b and c parameters of unit cells and the volume of unit cells decrease, and the crystal lattice becomes contracted.

The thermal stability of the CuTa_2−x_V_x_O_6_ solid solution was determined by performing DTA-TG tests of all single-phase samples for x = 0.00, 0.20, 0.25 and 0.30. Meanwhile, in the DTA curves of all samples, made up to 1500 °C, one endothermic effect was recorded, with an onset temperature in the range of 1270–1350 °C, depending on the composition of the sample. The temperature of the onset of the effects recorded for all samples was always lower than the melting point of CuTa_2_O_6_, and it decreased with the increasing degree of incorporation of V^5+^ ions. DTA curves of the tested samples are not included in this work due to the recorded very small thermal effect.

**Figure 2:**
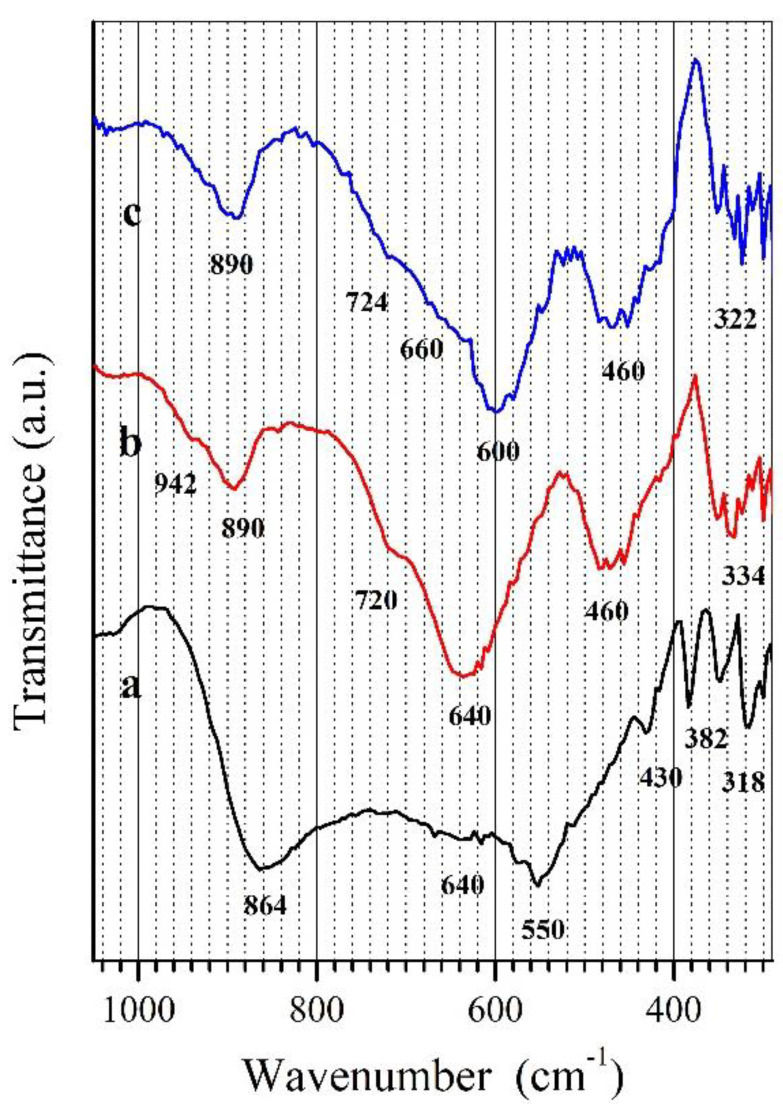
IR spectra of: (**a**) CuV_2_O_6_, (**b**) CuTa_1.70_V_0.30_O_6_ (x = 0.30), (**c**) CuTa_2_O_6_.

In order to characterize the properties more closely and confirm the structure of the obtained CuTa_2−x_V_x_O_6_ solid solution, IR spectra of all its single-phase samples (x = 0.2, 0.25 and 0.3) and the CuTa_2_O_6_ matrix were recorded. Figure 2 shows the IR spectra of CuV_2_O_6_ (curve a), CuTa_2−x_V_x_O_6_ solid solution for x = 0.3, with the highest degree of incorporation of V^5+^ ions into the CuTa_2_O_6_ crystal lattice (curve b) and the CuTa_2_O_6_ compound (curve c). High distortion of MO_x_ polyhedra characteristic for compounds of vanadium, tantalum and copper makes analysis of their IR spectra very difficult [45,46,47,48,49].

The IR spectrum of CuV_2_O_6_ comprised two broad absorption bands with maxima at 864 and 550 cm^−1^ (Figure 2, curve a). The absorption band with a maximum at 864 cm^−1^ can be ascribed to stretching vibrations of V-O bonds in distorted VO_6_ octahedra (characteristic for CuV_2_O_6_ structure) [48,50]. IR spectra of solid solution CuTa_1.70_V_0.30_O_6_ (x = 0.30), (Figure 2, curve b), and its matrix CuTa_2_O_6_ (Figure 2, curve c) contains four bands in the boundaries of 950–850 cm^−1^, 750–550 cm^−1^, 520–430 cm^−1^ and 380–300 cm^−1^ with maxima at about 890, 640, 460 and 330 cm^−1^. It follows from the literature survey that these bands are characteristic of solid tantalates (V) and vanadates (V) [48,50,51,52,53]. The similarity of these IR spectra corroborates that the solid solution adopts the CuTa_2_O_6_ structure. An intensive band with an absorption maximum registered in the region 950–850 cm^−1^ at ~870 cm^−1^ can be attributed to the stretching vibrations of Ta-O and V-O bonds in distorted TaO_6_ and TaO_7_ as well as VO_4_ and VO_6_ polyhedra [48,49,51,52,54]. Bands in the range of 750 to 550 cm^−1^ and 520–430 cm^−1^ can be attributed to the stretching vibrations of the Cu-O bonds in the distorted CuO_x_ polyhedra [45,46] and the stretching vibrations of M-O (M = Ta, V) bonds in the moderately distorted MO_7_, MO_6_ and VO_4_ polyhedra [48,49,50,51,52,53,54]. The bands registered in the range of 350 to 330 cm^−1^ can be attributed to bending vibrations of the O-M-O bridges (M = Cu, Ta, V), or they are of mixed character [48,49,50,53].

The comparative analysis of positions and intensities of absorption bands in IR spectra of CuTa_2_O_6_ and CuTa_2−x_V_x_O_6_ solid solution samples has revealed that incorporation of vanadium ions in the crystal lattice of CuTa_2_O_6_ mainly affects the position of the band with a maximum at 600 cm^−1^, which shifts gradually towards higher wavenumbers with an increase of vanadium content reaching 640 cm^−1^ in the spectrum of CuTa_2−x_V_x_O_6_ for x = 0.3 (Figure 2, curve d). The affection of the position of only one absorption band as a result of the increase of vanadium content seems to indicate that the crystal structure of the solid solution is built up of at least two crystallographically-independent polyhedra, and this less distorted one is preferentially occupied by vanadium ions.

**Figure 3:**
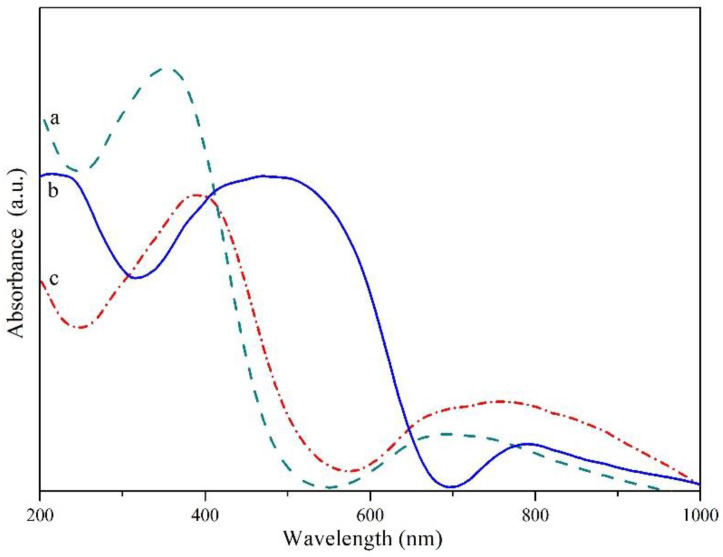
UV-Vis absorption spectra of: (**a**) CuTa_2_O_6_ (x = 0), (**b**) CuV_2_O_6_, (**c**) CuTa_1.70_V_0.30_O_6_ (x = 0.30).

In the next stage of works, in order to qualify the new phase obtained by the class of conductors, insulators or semiconductors, single-phase samples containing the CuTa_2−x_V_x_O_6_ solid solution were tested by the UV-Vis-DRS method. The UV-vis diffuse reflectance spectra were converted to absorbance spectra by the Kubelka-Munk method [55]. Figure 3 shows the Uv-Vis absorption spectra of compounds: CuTa_2_O_6_ (curve a), CuV_2_O_6_ (curve b) and a solid solution of CuTa_2−x_V_x_O_6_ for x = 0.30 (curve c).

**Figure 4:**
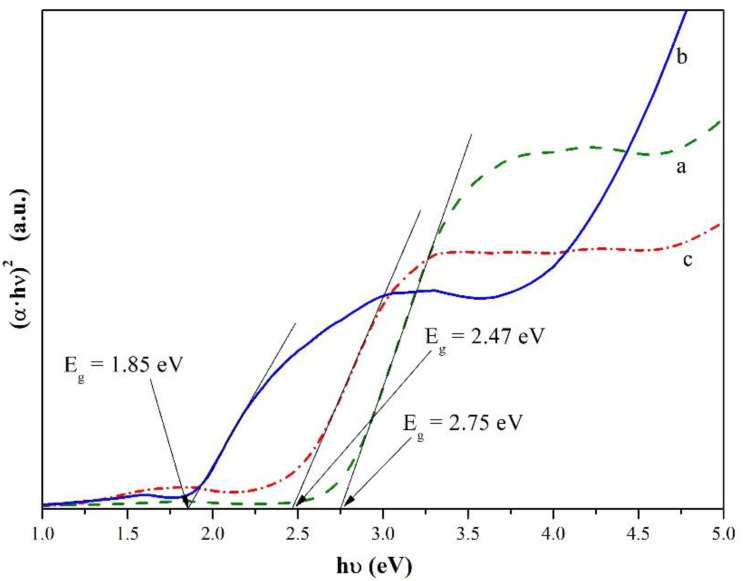
Estimated band gap energy of: (**a**) CuTa_2_O_6_ (x = 0), (**b**) CuV_2_O_6_, (**c**) CuTa_1.70_V_0.30_O_6_ (x = 0.30).

In addition, the values of the band gap energy were estimated for the CuTa_2_O_6_ and CuV_2_O_6_ compounds and the solid solution formed as a function of the degree of incorporation (x) of V^5+^ ions into the CuTa_2_O_6_ crystal lattice in place of Ta^5+^ ions. It was found that the value of the energy gap for CuV_2_O_6_ is 1.85 eV, and the energy gap for the CuTa_2−x_V_x_O_6_ solid solution ranges from 2.75 to 2.47 eV for 0.0 ≤ x ≤ 0.30 (Figure 4). These results show that the magnitude of the energy gap decreases with increasing the degree of incorporation of V^5+^ ions in the CuTa_2_O_6_ crystal lattice in place of Ta^5+^ ions.

The determined values of the band gap energy indicate that the new solid solution is a semiconductor.

### 3.2. Phase Equilibria in the Subsolidus Region of the CuO–Ta_2_O_5_–V_2_O_5_ System

**Figure 5:**
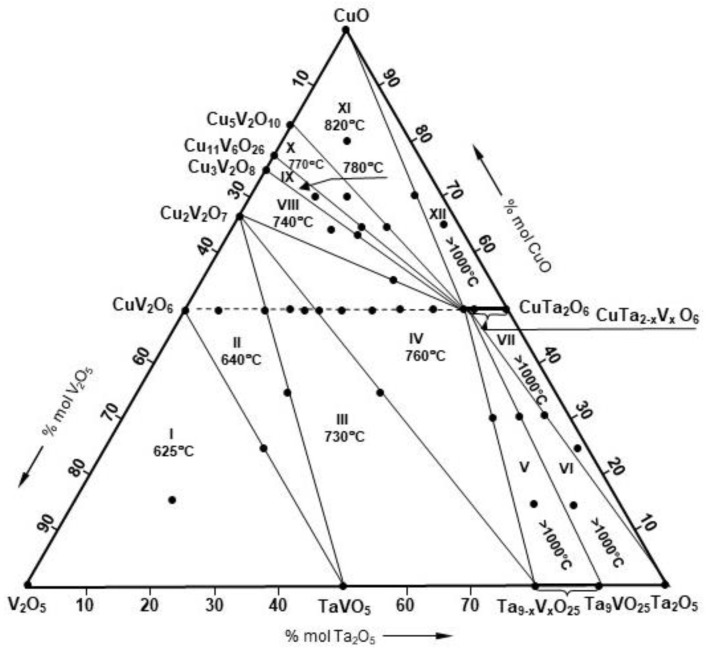
Division of the CuO–Ta_2_O_5_–V_2_O_5_ ternary system into partial subsystems.

The phase composition of the tested samples (1–10) after the last heating stage, i.e., in the state of equilibrium (Table 1), made it possible to distinguish seven partial subsystems in the CuO–Ta_2_O_5_–V_2_O_5_ system (Figure 5), i.e., I. V_2_O_5_–TaVO_5_–CuV_2_O_6_, II. CuV_2_O_6_–TaVO_5_–Cu_2_V_2_O_7_, III. Cu_2_V_2_O_7_–TaVO_5_–Ta_9_VO_25_, IV. Ta_9_VO_25_–CuTa_2_O_6(s.s.)_–Cu_2_V_2_O_7_, V. CuTa_2_O_6(s.s.)_–Ta_9_VO_25_, VI. CuTa_2_O_6_–Ta_9_VO_25_–Ta_2_O_5_, VII. Cu_2_V_2_O_7_–CuTa_2_O_6(s.s.)_.

The final verification of the subsolidus area of the CuO–Ta_2_O_5_–V_2_O_5_ system consisted in the preparation of additional samples (Nos. 11–30) constituting mixtures of these phases, which, based on the results of previous studies, were considered to be equilibrium and represent real two-component systems (samples containing two phases in a state of equilibrium) or partial systems (samples containing three phases in equilibrium). The compositions of these mixtures, as calculated for the components of the CuO–Ta_2_O_5_–V_2_O_5_ system, corresponding to the compositions of the samples, are presented in Table 4.

The prepared mixtures of phases were subjected to long-term heating at temperatures slightly lower (by ~20°) than the temperatures of the first effects recorded on their DTA curves. XRD analysis of these preparations showed that despite many hours of heating at temperatures close to the onset of melting, the phase composition of the samples did not change. This proves that the initial mixtures corresponded with their composition to the predetermined coexisting phases in the equilibrium state in individual systems.

The data presented in Table 3 not only confirmed the conclusions resulting from previous studies on the CuV_2_O_6_–CuTa_2_O_6_ binary system, but also allowed us to divide the CuO–Ta_2_O_5_–V_2_O_5_ system into twelve partial systems (Figure 5), i.e.,

1. V_2_O_5_–TaVO_5_–CuV_2_O_6_
2. CuV_2_O_6_–TaVO_5_–Cu_2_V_2_O_7_
3. Cu_2_V_2_O_7_–TaVO_5_–Ta_9_VO_25(s.s)_
4. Ta_9_VO_25(s.s.)_–CuTa_2_O_6(s.s.)_–Cu_2_V_2_O_7_
5. CuTa_2_O_6(s.s.)_–Ta_9_VO_25(s.s.)_
6. CuTa_2_O_6(s.s.)_–Ta_9_VO_25_–Ta_2_O_5_
7. CuTa_2_O_6(s.s.)_–Ta_2_O_5_
8. Cu_2_V_2_O_7_–CuTa_2_O_6(s.s.)_–Cu_3_V_2_O_8_
9. Cu_3_V_2_O_8_–CuTa_2_O_6(s.s.)_–Cu_11_V_6_O_26_
10. Cu_11_V_6_O_26_–CuTa_2_O_6(s.s.)_–Cu_5_V_2_O_10_
11. Cu_5_V_2_O_10_–CuTa_2_O_6(s.s.)_–CuO
12. CuO–CuTa_2_O_6(s.s.)_

On the basis of the DTA tests of samples after the last heating stage, the temperatures to which the phases representing individual partial systems and the relevant real two-component systems coexist in the solid state were determined. The temperatures of the onset of the first endothermic effect, recorded on the DTA curves of preparations in the state of equilibrium, and corresponding to a given partial system or a real two-component system, were assumed as these temperatures. The melting points of the mixtures of the phases coexisting in the given system are given in Table 5.

**Figure 6:**
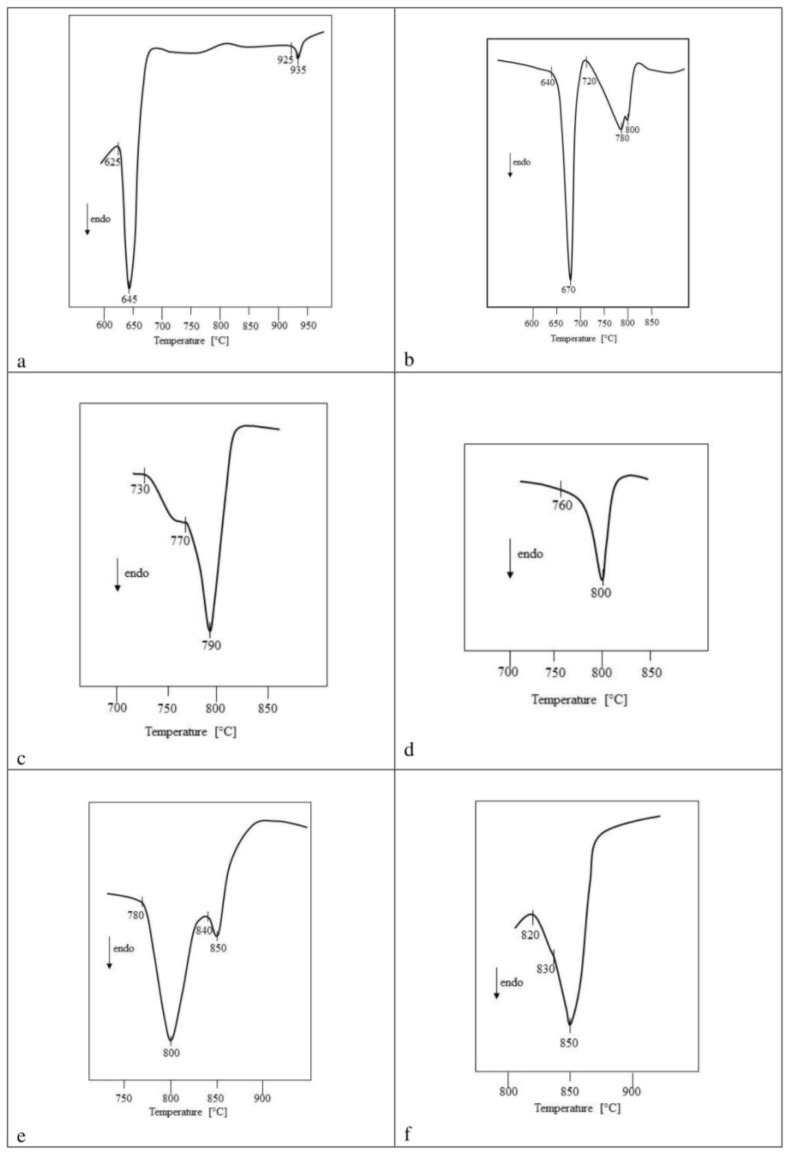
Fragment of DTA curves of samples containing coexisting phases: (**a**) CuV_2_O_6_, TaVO_5_, V_2_O_5_ (subsystem I), (**b**) CuV_2_O_6_, TaVO_5_, Cu_2_V_2_O_7_ (subsystem II), (**c**) TaVO_5_, Cu_2_V_2_O_7_; Ta_9_VO_25(s.s.)_ (subsystem III), (**d**) Cu_2_V_2_O_7_, CuTa_2_O_6(s.s.)_, Ta_9_VO_25(s.s.)_ (subsystem IV), (**e**) Cu_3_V_2_O_8_, CuTa_2_O_6(s.s.)_, Cu_11_V_6_O_26_ (subsystem IX), (**f**) Cu_5_V_2_O_10_, CuTa_2_O_6(s.s.)_, CuO (subsystem XI).

The DTA curves of selected samples recorded in the air atmosphere up to 1000 °C are shown in Figure 6.

The presented DTA curves of selected samples in the equilibrium state showed, among others, that the partial V_2_O_5_–TaVO_5_–CuV_2_O_6_ system (Figure 6a) melts eutectically at a temperature of 625 ± 5 °C, which means that its components coexist in the solid state to this temperature. Determining the composition of the triple eutectic mixture formed in this constituent system is beyond the scope of this work and will be determined in future studies.

In this part of the study, it was also established that the components of the second partial system CuV_2_O_6_–TaVO_5_–Cu_2_V_2_O_7_ coexist in the subsolidus area up to the temperature of 640 ± 5 °C (Figure 6b). This temperature is related to the smelting of the eutectic mixture formed between CuV_2_O_6_ and Cu_2_V_2_O_7_ in the two-component V_2_O_5_–CuO system [13,14,15,16,17,18,19,20,21].

Based on the DTA curve of a sample containing TaVO_5_ at equilibrium with Cu_2_V_2_O_7_ and with a saturated solid solution of Ta_9−x_V_x_O_25_ (Figure 6c), it was found that these phases coexist in the solid state up to 730 ± 5 °C. In this case, it is related to the melting of the TaVO_5_ eutectic mixture with Cu_2_V_2_O_7_.

Moreover, it was found that the solid solution obtained as part of this work, with the structure of CuTa_2_O_6_ and the maximum degree of incorporation of V^5+^ ions, remains at equilibrium in the solid state with Ta_9−x_V_x_O_25_ and Cu_2_V_2_O_7_, as well as Cu_2_V_2_O_7_ and Cu_3_V_2_O_8_ up to 760 and 740 ± 5 °C, respectively (Figure 6d). The melting point of subsystem IV is related to the congruent melting of Cu_2_V_2_O_7_, and the VIII subsystem to the melting of the eutectic mixture formed between Cu_2_V_2_O_7_ and Cu_3_V_2_O_8_ [15,21].

It was additionally established that CuTa_1.7_V_0.3_O_6_ coexists in the subsolidus region with Cu_3_V_2_O_8_ and Cu_11_V_6_O_26_ to ~780 °C (Figure 6e) and with Cu_11_V_6_O_26_ and Cu_5_V_2_O_10_ to ~770 °C, while with Cu_5_V_2_O_10_ and CuO up to 820°C (Figure 6f). These results indicate that the fields IX and XI of the tested ternary oxide system V_2_O_5_–CuO–Ta_2_O_5_ (Figure 5) melt peritectically but X eutectically. It is related to both the incongruent melting of Cu_3_V_2_O_8_ and Cu_5_V_2_O_10_ compounds and the eutectic formed between Cu_11_V_6_O_26_ and Cu_5_V_2_O_10_ [15,20,26].

The exact melting points of the partial systems V-VII and XII were not determined because no thermal effects were recorded on the DTA curves of samples in equilibrium state, representing these fields, in the temperature range up to 1000 °C.

Nevertheless, on the basis of such observation, it can be stated without doubt that the mixtures of components of the mentioned subsystems melt above 1000 °C.

The results of these studies are of great practical importance, including when designing new multi-component materials, such as, e.g., catalysts. They provide important information on the type of phases formed in the CuO–V_2_O_5_–Ta_2_O_5_ system, and at what temperatures these phases coexist with each other in the solid state, i.e., their mixtures can be used without the risk of melting and the associated change in their composition.

## 4. Conclusions

The research results obtained as part of this work authorize the following conclusions:

1. In the three-component system of CuO–V_2_O_5_–Ta_2_O_5_ oxides, a substitution solid solution is formed with a limited range of homogeneity and the general formula CuTa_2−x_V_x_O_6_ for 0 < x ≤ 0.3.
2. The new solid solution is formed by the incorporation of V^5+^ ions in the CuTa_2_O_6_ crystal lattice in place of Ta^5+^ ions. The maximum degree of V^5+^ ion incorporation is 15 mol%.
3. CuTa_2−x_V_x_O_6_ for 0 < x ≤ 0.3 crystallizes in the tetragonal system, and with the increase of the degree of incorporation of V^5+^ ions in place of Ta^5+^ ions into the CuTa_2_O_6_ crystal lattice, the parameters a = b and c and the volume of unit cells decrease, and the crystal lattice contracts.
4. The CuTa_2−x_V_x_O_6_ solid solution is stable, depending on its composition, from a temperature of 1350 °C for x = 0.00 to 1270 °C for x = 0.30.
5. The IR spectra of solid solution CuTa_2−x_V_x_O_6_ (0 < x ≤ 0.30) and its matrix CuTa_2_O_6_ are very similar what corroborates their isostructural character. IR spectra of these phases contain bands in the boundaries of 950–850 cm^−1^, indicating that crystal lattices of these phases are built up of considerably distorted polyhedra. The incorporation of vanadium ions in the crystal lattice of CuTa_2_O_6_ mainly affects the position of the band with a maximum at 600 cm^−1^, which shifts gradually reaching 640 cm^−1^ in the spectrum of CuTa_2−x_V_x_O_6_ for x = 0.30.
6. The CuTa_2−x_V_x_O_6_ solid solution is a semiconductor, and the value of the energy gap for the solid solution ranges from 2.75 to 2.47 eV for 0.00 ≤ x ≤ 0.30.
7. The three-component system of metal oxides CuO–V_2_O_5_–Ta_2_O_5_ consists of 12 partial systems, i.e., I. V_2_O_5_–TaVO_5_–CuV_2_O_6_, II. CuV_2_O_6_–TaVO_5_–Cu_2_V_2_O_7_, III. Cu_2_V_2_O_7_–TaVO_5_–Ta_9_VO_25(s.s)_, IV. Ta_9_VO_25(s.s.)_–CuTa_2_O_6(s.s.)_–Cu_2_V_2_O_7_, V. CuTa_2_O_6(s.s.)_–Ta_9_VO_25(s.s.)_, VI. CuTa_2_O_6(s.s.)_–Ta_9_VO_25_–Ta_2_O_5_, VII. CuTa_2_O_6(s.s.)_–Ta_2_O_5_, VIII. Cu_2_V_2_O_7_–CuTa_2_O_6(s.s.)_–Cu_3_V_2_O_8_, IX. Cu_3_V_2_O_8_–CuTa_2_O_6(s.s.)_–Cu_11_V_6_O_26_, X. Cu_11_V_6_O_26_–CuTa_2_O_6(s.s.)_–Cu_5_V_2_O_10_, XI. Cu_5_V_2_O_10_–CuTa_2_O_6(s.s.)_–CuO, XII. CuO–CuTa_2_O_6(s.s.)_.

## Figures and Tables

**Table 1:** The composition of initial mixtures and the results of phase XRD analysis of the samples from the CuV_2_O_6_–CuTa_2_O_6_ system after the last heating stage.

No.	Composition of Initial Mixtures [Mol%]		Composition of Initial Mixtures Calculated as Oxides [Mol%]			Final Heating Temperature [°C]	Composition of Samples at Equilibrium
	CuV_2_O_6_	CuTa_2_O_6_	CuO	V_2_O_5_	Ta_2_O_5_		
1	90.00	10.00	50.00	45.00	5.00	620	CuV_2_O_6_, TaVO_5_, Cu_2_V_2_O_7_
2	75.00	25.00	50.00	37.50	12.50		Cu_2_V_2_O_7_, TaVO_5_
3	66.67	33.33	50.00	33.33	16.67	720	Ta_9_VO_25(s.s.)_, Cu_2_V_2_O_7_, TaVO_5_
4	63.64	36.36	50.00	31.82	18.18		
5	60.00	40.00	50.00	30.00	20.00	700	CuTa_2_O_6(s.s.)_, Ta_9_VO_25(s.s.)_, Cu_2_V_2_O_7_
6	50.00	50.00	50.00	25.00	25.00		
7	40.00	60.00	50.00	20.00	30.00	725	
8	33.33	66.67	50.00	16.67	33.33		
9	20.00	80.00	50.00	10.00	40.00		
10	10.00	90.00	50.00	5.00	45.00	900	CuTa_2_O_6(s.s.)_

**Table 2:** The composition of initial mixtures and the results of phase XRD analysis of additional samples from the CuTa_2_O_6_–CuV_2_O_6_ system at equilibrium state.

No.	Composition of Initial Mixtures [Mol%]		Composition of Initial Mixtures Calculated as Oxides [Mol%]			Parameter X in the Obtained Solid Solution with Formula CuTa_2−x_V_x_O_6(s.s)_	Composition of Samples at Equilibrium
	CuV_2_O_6_	CuTa_2_O_6_	CuO	V_2_O_5_	Ta_2_O_5_		
1.	10.00	90.00	50.00	5.00	45.00	0.20	CuTa_1.80_V_0.20_O_6_
2.	12.50	87.50	50.00	6.25	43.75	0.25	CuTa_1.75_V_0.25_O_6_
3.	15.00	85.00	50.00	7.50	42.50	0.30	CuTa_1.70_V_0.30_O_6_
4.	17.50	82.50	50.00	8.75	41.25	0.30 (theoretical 0.35)	CuTa_1.70_V_0.30_O_6_, Cu_2_V_2_O_7_, Ta_9_VO_25_

**Table 3:** Unit cell parameters and volumes for the CuTa_2_O_6_ (x = 0) and solid solution CuTa_2-x_V_x_O_6_ for x = 0.20, 0.25 and 0.30.

x	a = b [nm]	c [nm]	V [nm^3^]	d_cal_. [g/cm^3^]	d_exp_. [g/cm^3^]
0.00	7.5221	3.7582	212.64643	8.14355	7.9775
0.20	7.5188	3.7572	212.40336	7.74633	7.9368
0.25	7.5172	3.7568	212.29037	7.64876	7.8163
0.30	7.5150	3.7560	212.12095	7.5531	7.5088

**Table 4:** The compositions of the initial mixtures of oxides and the results of the XRD analysis of the samples after the last heating stage.

No.	Composition of the Initial Mixtures [Mol%]			Final Stage of Heating [°C]	Phase Composition of the Samples in Equilibrium State
	CuO	V_2_O_5_	Ta_2_O_5_		
11.	15.00	70.00	15.00	620	CuV_2_O_6_, TaVO_5_, V_2_O_5_
12.	25.00	50.00	25.00	625	CuV_2_O_6_, TaVO_5_
13	35.00	41.00	24.00	725	Cu_2_V_2_O_7_, TaVO_5_
14.	35.00	23.00	42.00		Cu_2_V_2_O_7_, Ta_9_VO_25(s.s.)_
15.	30.00	14.00	56.00	900	CuTa_2_O_6(s.s.)_, Ta_9_VO_25(s.s.)_
16.	15.00	13.00	72.00	900	CuTa_2_O_6(s.s.)_, Ta_9_VO_25(s.s.)_
17.	30.00	9.00	61.00	900	CuTa_2_O_6(s.s.)_, Ta_9_VO_25_
18.	15.00	7.00	78.00	900	Ta_9_VO_25_, CuTa_2_O_6(s.s.)_, Ta_2_O_5_
19.	30.00	4.00	66.00	900	CuTa_2_O_6(s.s.)_, Ta_2_O_5_
20.	25.00	2.00	73.00	900	CuTa_2_O_6(s.s.)_, Ta_2_O_5_
21.	54.00	11.00	35.00	725	Cu_2_V_2_O_7_, CuTa_2_O_6(s.s.)_
22.	65.00	25.00	10.00	725	Cu_2_V_2_O_7_, CuTa_2_O_6(s.s.)_, Cu_3_V_2_O_8_
23.	64.00	16.00	20.00	725	Cu_3_V_2_O_8_, CuTa_2_O_6(s.s.)_
24.	70.00	18.00	12.00	750	Cu_3_V_2_O_8_, CuTa_2_O_6(s.s.)_, Cu_11_V_6_O_26_,
25.	65.00	15.00	20.00	750	Cu_11_V_6_O_26_, CuTa_2_O_6(s.s.)_
26.	70.00	13.00	17.00	750	Cu_11_V_6_O_26_, CuTa_2_O_6(s.s.)_, Cu_5_V_2_O_10_
27	65.00	12.00	23.00	750	Cu_5_V_2_O_10_, CuTa_2_O_6(s.s.)_
28	80.00	10.00	10.00	750	CuO, Cu_5_V_2_O_10_, CuTa_2_O_6(s.s.)_
29	70.00	5.00	25.00	900	CuO, CuTa_2_O_6(s.s.)_
30	65.00	3.00	32.00	900	CuO, CuTa_2_O_6(s.s.)_

**Table 5:** Melting point of the samples in equilibrium state.

No.	Phases at Equilibrium	Melting Point [°C]
1.	V_2_O_5_–TaVO_5_–CuV_2_O_6_	625
2.	CuV_2_O_6_, TaVO_5_	650
3.	CuV_2_O_6_–TaVO_5_–Cu_2_V_2_O_7_	640
4.	Cu_2_V_2_O_7_, TaVO_5_	730
5.	Cu_2_V_2_O_7_–TaVO_5_–Ta_9_VO_25(s.s.)_	730
6.	Cu_2_V_2_O_7_–Ta_9_VO_25(s.s.)_	760
7.	Ta_9_VO_25(s.s.)_–CuTa_2_O_6(s.s.)_–Cu_2_V_2_O_7_	760
8.	CuTa_2_O_6(s.s.)_–Ta_9_VO_25(s.s.)_	>1000
9.	Cu_2_V_2_O_7_–CuTa_2_O_6(s.s.)_	760
10.	Cu_2_V_2_O_7_–CuTa_2_O_6(s.s.)_–Cu_3_V_2_O_8_	740
11.	Cu_3_V_2_O_8_–CuTa_2_O_6(s.s.)_	750
12.	Cu_3_V_2_O_8_–CuTa_2_O_6(s.s.)_–Cu_11_V_6_O_26_	780
13.	Cu_11_V_6_O_26_–CuTa_2_O_6(s.s.)_	780
14.	Cu_11_V_6_O_26_–CuTa_2_O_6(s.s.)_–Cu_5_V_2_O_10_	770
15.	Cu_5_V_2_O_10_–CuTa_2_O_6(s.s.)_	810
16.	Cu_5_V_2_O_10_–CuTa_2_O_6(s.s.)_–CuO	820
17	Ta_9_VO_25(s.s.)_–CuTa_2_O_6(s.s.)_–Ta_2_O_5_	>1000
18.	CuO–CuTa_2_O_6(s.s.)_	>1000
19.	CuTa_2_O_6(s.s.)_–Ta_2_O_5_	>1000

## Data Availability

Data sharing is not applicable to this article.
